# Treatment of eating disorders: voices from a ward

**DOI:** 10.1080/17482631.2021.1983948

**Published:** 2021-10-11

**Authors:** Cathrine Solhaug Storli, Sissel Alsaker

**Affiliations:** a Social Educator in Mental Health, Crisis Resolution Team, Helgeland Hospital, Mo I Rana, Norway; b Department of Mental Health, Faculty of Medicine and Health Science, NTNU-Norwegian University of Science and Technology, Trondheim, Norway

**Keywords:** Mental health, eating disorders, patients, treatment, experiences

## Abstract

**Purpose:**

The purpose of this study is to explore and develop knowledge about treatment experiences of people suffering from severe eating disorders, by highlighting the patient’s perspective in treatment. The study’s issue is: “How do patients with severe eating disorders experience everyday hospital/in-hospital treatment, and how do they value the impact of their experiences in treatment?”

**Method:**

The study takes a qualitative approach, where patients wrote diaries that formed the data. There were 3 participants who wrote diaries twice a day for 2 weeks. In this study, thematic interpretative analysis of narrative data was used.

**Results and conclusion:**

The results of this study indicate that several health-related experiences emerged during treatment. The results show that the experiences of in-hospital treatment for severe ED are mostly characterized by experiences that might reduce the participants' sense of meaning. The study suggests new arenas (besides taking control over food/their bodies) for individual patients experienced mastery in their own recovery process.

## Introduction

Eating disorders (ED) is one of the most dominant issues in terms of mental health after puberty age (Det Kongelige Helse- og omsorgsdepartementet, 2014-2015, p. 155) and an increasing number of people seek help from healthcare services (Skårderud et al., 2004). Torgersen (2016) estimated that close to 50,000 women and men in Norway suffer from either overeating, bulimia or anorexia and additionally several people that are not registered with a diagnosis (Norsk helseinformatikk, 2017). We do not know the exact number of people suffering from ED worldwide, but we know that there are differences due to socio-cultural factors and that there are more people suffering from it in western countries (Hoek, 2016). Persons who suffer from ED experience challenges related to meals, thoughts and feelings affecting their every day and mental health (Skårderud, 2000). How we understand the meaning of behaviour related to ED is debated. However, their behaviour is often characterized by a need to experience control, where their bodies become an affective regulator for psychological factors (Skårderud et al., 2012). Some patients therefore experience utility functions of the ED as they may have great knowledge about food, but reduced understanding of how the food works for their body (Aase, 2004).

In treatment, the goals for the National health- and hospital plan (Norway) are that the patient’s needs should be the focus for the services that are given (Holter, 2017). Further, Karlsson and Nevonen (2012) found that the patient’s experience of being seen and met is essential for the treatment process, and it is argued that health promoting measures through increased mastery and wellness can contribute to a better mental health (Worlds Health Organization (WHO), 2014) as well as an overall recovery-process (McArdle & Sheridan, 2016).

Following from the argument above, we contend that treatment of people with ED is a great challenge because the sparse we know of how to help people suffering with this disorder.

More professional training is needed when it comes to increasing knowledge about symptoms and effective treatment (Hart et al., 2011), personalizing and adjusting the therapy objectives and timing related to cognitive functioning (Grau et al., 2019).

Treatment guidelines for ED have weaknesses due to their limited representation of the patients’ values and preferences in treatment (Helsedirektoratet, 2017). Several patients do not experience a better life after treatment (Modum Bad, 2016) as their weight may be normalized but their mental health is not experienced as improved.

Regarding the number of people that suffer from ED and the impact the disorder has upon their mental health and everyday living, this study answers the call for increased knowledge about patients’ experiences and how these impact them during everyday/in-hospital treatment.

## Method

### Recruiting and sample

With the desire to increase knowledge about patients experiences during treatment for severe ED, we recruited participants who were in-hospital treatment in Norway. The hospital’s department manager and the Head of Section invited patients to participate and gave them a letter of information (prepared by the researchers) containing advantages and disadvantages the participants might experience during the data collection period of 2 weeks. The study had 4 participants to begin with, but because one resigned the study had 3 participants.

The participants were within the age group from 18 to 30 years, they were all at an in-hospital treatment for severe ED. What kind of ED they had or their sex was not known to the researchers, due to the researcher not receiving any identifiable information about them.

Some of the criteria for in-hospital treatment for severe ED can be a large weight loss, unstable somatic parameters or absence of effect by outpatient treatment. The treatment department was small, with few in-hospital patients. Interdisciplinary treatment teams evaluated the patient’s condition. The treatment was divided into different phases. The first week was called a “trying week”, where patients could get to know the treatment facility and evaluate their motivation for further treatment. The staff also evaluated if the treatment was suitable for individual patients. If the patients wanted to continue treatment after this week, a treatment contract was signed. The treatment was based on the type of ED; anorexia nervosa, bulimia nervosa or overeating disorder. The hospitals’ main goal is to add frameworks, structures, insights and experiences to their patients. At in-hospital treatment there are predictable weekly plans. The treatment programme contains individual psychotherapy, psychoeducational therapy, milieu therapy and assessment of the need for medical treatment. Some of the therapy was group based with close follow-up by the health staff regarding meals because re-nutrition and challenges related to anxiety and food was important elements of the environmental therapy.

### Ethics

All participants participated voluntarily and signed an informed consent form. The study was approved by NSD (Norwegian Centre for Research Data), the treatment facility’s local management and reported to the hospital's Data Protection Official. Patients from the hospital were invited to participate after the first week of hospital treatment ended, due to the treatment programmes establishing phase where this first week were used to evaluate if they wanted to go through with the treatment. The researchers did not meet with or receive any identifiable information about those who agreed to participate.

### Data collection

With the wish to get a novel perspective on patients’ experiences during treatment for severe ED, diaries were chosen for data collection. This method was chosen regarding research ethics, as the participants could be in a vulnerable state due to ongoing treatment and with a hope that diaries could be a “safe space” for the participants to share their experiences.

By choosing this method, the participants could describe their experiences and reflections over a period of 2 weeks and gave the researchers primary data (Drageset and Ellingsen, 2009) by an insight of immediate experiences (Holloway, 2008). The diaries were marked with alphabetic letters, providing no identifiable information about the participants. The participants wrote diaries two times a day, for 14 days, during treatment, answering two open questions a day: In the morning “How do you think this day will be?” and later on “How did you experience the day?”. On the last day of participating, they were given three questions: “How did you experience writing a diary?”, “Can you describe what you think is positive and negative during the period of treatment?” and “Can you describe what you are experiencing in treatment, that may help you after treatment?” All participants completed writing diaries for 14 days. The transcribed documents extend from the diaries, and were a total of 35 pages (13,100 words).

### Data analysis

In line with our aim, we searched for experiences that gave a sense of meaning to the individual participant during treatment, and an interpretive approach and a thematic analysis of the narratives was conducted.

The first author was responsible for the analysing the data material, where this researcher searched for the participants’ expressions of meaning in the transcripts, and how these experiences of meaning were related to their everyday life (Josephsson & Alsaker, 2015) during treatment. By reading the participant diaries and interpreting these, the results include both the narrative discourse (the participants' written words) and the researchers' understanding/interpretation of their written descriptions (Jacobsson, Lappalinen, Meeuwise & Sward, 2015).

The analysis was performed in four steps. 1) Transcription of the diaries (sensitive information was deleted/made anonymous); 2) Reading transcripts for each diary (while the researcher made note of immediate thoughts and number these); 3) Categorizing and studying common themes; and 4) Writing up the narrative stories.

In more detail each diary was transcribed into individual text documents. The main themes were found by coding each element with a letter, in the individual participants transcribed document. For example, K in the front of sentences where the participants described situations complicated, and L for eventual descriptions that were described to resolve the situation. Further, these letters had a colouring code. All sentences in the documents were coded. The first author then studied eventual commonalities regarding repetitive experiences. If and when several participants described similar experiences on one theme, the researchers could pair various sections from the individual diaries that were about a common theme, in a new document.

Therefore, the main themes and sub themes were identified as they were found to express the underlying meaning through condensed meaning units and forming codes/categories on the research interpretative level (Graneheim & Lundman, 2004). The participants' text became the starting point of new stories (narrative stories) formed by the researcher.

The study’s results show three main themes mediating the patients’ experiences during treatment for severe ED and related to the researcher’s interpretation as well as to existing literature.

## Results

The study’s results will now be shown through three narrative themes as follows: 1) Dealing with changes 2) To be something other than a patient, 3) Letting go of the ED. The participant quotes are shown in cursive writing.

### Dealing with changes

Participants expressed challenges regarding if and how nutrition and their bodies were linked.

Some expressed the need for motivation to succeed in eating their meals during treatment.

*“I was determined that I wouldn`t let myself discuss with my ED to do or don`t do the work”*. The participants still described that their bodily changes were difficult to accept.

*“It was a little difficult to look at myself in the mirror, lately this have been difficult, difficult to see and feel that my body is changing”.*

*“The despise of bodily curves are chasing me now as I gain weight just gets more hurtful to accept. Painful thoughts of my body are consuming me”.*

The participants expressed reduced motivation to gain weight because of their own bodies and not understanding their bodies need for food.

*My body should have gained more energy now than it had before because I´ve gained this much weight. But instead, I experience that my body gets heavier and more and more tired- in line with gaining weight. It seems so backwards.*

*“Sometimes I`m striving to accept and understand the need for the amount of food, and to allow myself to taste it”*. It was described as especially difficult to maintain the meal plan, because it experienced positive sides of behaviour characterized by the ED. *“I’m* longing *to use the meals as a regulator for hurtful thoughts, feelings, memories and reminders”.*

In a way, it seemed like the ED had an escape function. “*It`s easier to escape to the ED, not having to cope with this”.*

During treatment both meal-plans and days of weighing were used as tools for the health staff to systematize and measure progress. Some described a challenging change in focus during treatment, related to the measures.

*I`ve never been so obsessed with weight at home, although I had control by food, amount and the content of food. I experience this difficult because I`m more obsessed of my weight now than ever, and I`m tired of this ongoing nagging about weight- and BMI.*

Measures of progress in relation to weight were also contributing to consequences, no matter the outcome.

*It’s difficult no matter what the number shows. If I´ve gained a lot it gets tough, if I´ve gained less than what I´m supposed to I have to add more to my list of meals and I don´t want that.*

Some had already struggled to complete their meal lists and described anxiety about eventual consequences if the amount of food on their meal plan increased.

*“I feel ashamed when I don`t complete meals, then I just want to hide and give up because I feel so unsuccessful”*. But some also described it as helpful eating with others *“Lunch today was better; I sat down and had a relaxed conversation with a previous patient as while we ate”.*

One of the participants was sent home from the hospital for a so-called “week of considering”, because of the results of weighing that showed a weight loss of 0.7 kg. “A week of considering” is as a decision made based on eventual agreements that are not complied by the patient (Husom, 2003).

*I think it´s difficult to go home for a ‘week of considering’ now, because I´m struggling with motivation and faith in myself at this time. At the same time, difficult thoughts and reminders is provoked by spending time at my place of home, because I have painful memories* from this place.

While at home, this participant described that destructive actions were taken, trying to get a “break”. “*After breakfast I threw up, because I felt a need to «relieve the pressure. Calm myself down to avoid an intense feeling of being full*”. And further: *“My mood is not good now. I experience low energy, a feeling of discouragement and hopelessness, and that I easily get irritated. This stresses me”*. The participant expressed a wish to contact the staff at the hospital, but described

*I`ve debated with myself, worried a lot about the discharge from the hospital, when I´m going home to friends and family again. This forever ongoing pondering lead to a breakdown, and I of course didn`t manage to call the hospital*

### Discussion of “Changes”

The participants seemed to have an ongoing battle within themselves during treatment because of the experienced changes. Some tried motivating themselves several times to complete meals, by not discussing alternatives. As we understand it, the ED affects the participants' thought patterns, and in some way has an inner “voice”.

The littarature explains that people who suffers from ED can show reluctance to reduce symptoms because symptoms such as food restrictions or weight loss can contribute to positive feelings (Skårderud, Rosenvinge & Gòtestam, 2004).

In our understanding, the meals seemed to be challenging due to the participants experiences of being full and the absence of positive effects by completing meals. Further, meals contributed to difficult experiences such as self-loathing, because of the bodily changes that we interpret was reinforced by weighing, nutrition and mirror reflections.

Skårderud (2000) explains that weighing permits an observation of the patient's BMI, in relation to normal values. Weighing could also contribute to designing the meal plan. Aase (2004) found that during treatment for ED, the meals could be seen as a “prescription”, where the food is the “medication”, and that people who suffers from ED often lacks an understanding of how the food works for their body. Grau et al. (2019) points out that a cognitive impairment is more frequent for people with long-term ED (and malnutrition)—especially related to perceptual measures and non-verbal memories. Skårderud et al. (2012) argues that for patients with ED, their mind becomes the body and that these people may have reduced ability regarding mentalization, because good mentalization is related to the ability to regulate emotions.

Our results are in line with this by the fact that the participants mirror reflection no longer accompanies how they feel inside and thereby their bodies cannot be used in the ordinary way to communicate this during treatment. If the participants' experience of their own body is based on their outer- self, such as mirror-reflection, they may lack contact with their bodily signals. This is also found by Brogna and Caroppo (2010) by that for some with ED, the body can be viewed as an object and tool for communicating with others, and it can accompany what they`re feeling inside. Existing knowledge shows that when the body is used for coping with feelings of such factors, the body can become an affective regulator (Skårderud et al., 2012). If the meals previously have given the participants a chance to regulate/mute inner unease (Torgersen, 2016), then ED has been a way of coping with feelings (Aase, 2004), they may experience an inner unease during treatment. Following Vrabel (2014) the patients may not have acquired methods other than behaviour characterized by the ED, to cope with difficult feelings/thoughts. If this is the case for the participants in our study, they may have experienced that the ED was of help to them. Participants may have a reduced ability when it comes to regulating emotions. Their limited experience of inner safety and ability to self-regulate can contribute to a feeling of being inadequate, unsuccessful and out of control (Skårderud et al., 2012), so if they previously used their bodies to achieve control of the bodily changes may now be a confirmation that he/she is no longer in control. If the participants in this study did not possess other coping strategies for handling difficult thoughts/emotions than behaviour characterized by ED, this may challenge their experience of food giving positive effects when looking at their mirror reflection.

One participant specifically described experiences of consequences of weight, when being sent home from the hospital for a “week of considering”. The decision of sending this participant home for a “week of considering”, we interpret may have been the health-staff’s attempt to increase the motivation and engagement for further treatment. Because this participant expressed mental challenges by going home, it seems for us as the participants experienced needs is not heard. If so, this decision contradicts the goals of the National Health- and Hospital Plan, when it comes to the importance of having the patients’ needs as the main focus (Holter, 2017). At home, destructive actions were taken. Such destructive actions characterized by the ED, can be an attempt to mute an experienced physical discomfort (Skårderud, 2000). Possibly, this participant experienced that this was necessary to achieve an increased feeling of calmness at home. The participants clearly described more difficult than good experiences at home. In our understanding, it might be questioned if this week contributed to increased motivation at this point in treatment due to the participants' ambivalent motivation.

Torgersen (2016) found that different triggering factors might be the starting point for developing the ED, or maintaining it. We contend that if this is the case for this participant, being at home could lead to difficult experiences that might complicate maintaining meal structure during this week, as well as contributing to a feeling of not being seen, heard and/or understood by the health services and further affect motivation for treatment.

Here, we may point towards the knowledge of therapeutic alliance, where patients feel seen, met and understood as essential to the treatment process (Karlsson & Nevonen, 2012), as well as trust in the health staff, something that is found to be of great importance in treatment (Karlsson & Nevonen, 2012). This participant may experience that the health staff is more focused on the weight or illness than the psychological challenges, something that might impact the general treatment experience. Further, this may lead to the health services overlooking possible underlying causes of ED, such as predisposing or triggering conditions such as negative self-image, trauma, loss/conflict, bullying, etc. (Folkehelseinstituttet, 2009). It is recommended to focus on the patient’s mastery and health promoting work, rather than their illness during the treatment (McArdle & Sheridan, 2016). This is by seeing the humans behind the illness, how they experience meaning, mastery and motivation during treatment. Further, it is possible that the participants then increasingly could have experienced a greater sense of mastery and ownership in their own recovery process.

### To be something else than a patient

The participants described a longing for activities that could help them stay motivated during treatment. There were several expressions that described psychological challenges when they didn`t have much to do.

Sometimes *I get afraid and wonder about the meaning of life and the feeling that I´m throwing the life I´ve gotten away. Such thoughts usually come and are most intense at difficult days with little to do when I have much time alone to think*. As it was earlier this week; *got a strong urge to act destructively/escape by previous strategies, such as self- harming and taking cold showers, but managed to communicate with my contact who works here to derive this by playing cards*. I experienced *a useful and positive conversation with this contact*

It is positive that the *treatment does focus on other things, activities that complements the everyday- life here. Especially ‘healthy’ things, that`s not influenced by the ED. But to be honest I wish there was more to do than what`s planned for us. I get so frustrated when I have nothing to do. I just want to sleep all the time or be in physical activity- just to get away from all the difficult thoughts. Little to do means more time to think*

*I feel pretty tired of being in- hospitalized. I would like to have outpatient- treatment, but the team around me says it too early and that I must wait to get better. I think it`s difficult to be patient. The hours go by so slow, and I don`t know what to do*

*“I hope we`ll do something as a group today, it helps me handle the days here better and it feels meaningful”.*

In our interpretation we understand the participants expressions as a call for activities that were not focused on the ED, they never asked specifically for physical activity.

*“One of the staff took me out for a car ride, then we walked beside the ocean. It was good to see other things and talk about everyday- themes, as for example, the starry sky”*.

*It was nice to have a day where I could experience myself as something else than a patient in need of support and help. Got a feeling of being something more than an illness. It was a little painful to see how much the ED has taken from me when it comes to quality of life and how damaging it is, but it can be useful to remind myself of this when I feel like giving up the battle to become healthy*

Despite positive experiences due to activities the participants described missing being independent.

“*I miss just being me, not at patient in need of help and support”*. And those rules were frustrating; “*because some of the health staff are very strict, something I find challenging. Some of those who have worked here for several years are not that obsessed be the rules”*. The thought of experiencing more independence seemed to motivate. “*I believe that the treatment will be easier when I get more responsibility, more possibilities to go outside and experience more freedom”.*

### Discussion of “To be something else than a patient”

The participants described several experiences that we understood was of meaning for them during treatment. Conversations with the health staff, within groups, and with her dependents (family/relatives) were appreciated. Authors point towards that conversations with the health staff may contribute to a supportive atmosphere (Karlsson & Nevonen, 2012). From our analysis we found that because participants describe positive experiences through such conversations, it is likely that the health staff has succeeded in creating a good therapeutic alliance. Skårderud (2000) argues that conversations in groups are experienced positively, because patients can convey hope, similarity, receive/give advice, within an accepting arena. Skårderud further contends that conversations with the patients’ dependents and a therapist may contribute to a feeling of safety. To help maintain and promote relations as family and friends can be of importance in relation to the patient’s motivation and bettering their everyday life (Helsedirektoratet, 2017).

Although conversations were found to be of positive meaning, our results show that participants experienced too much time alone during the treatment, experiencing difficult thoughts. It seems like activities mean a lot for their motivation during the treatment, as they described that activities reduced difficult thoughts, prevented destructive behaviour and contributed to feeling more as a person, rather than an illness in need of help. The feeling of being a patient in need of help seemed to be amplified by having to maintain rules within the hospital, especially if the rules were held differently by the staff.

Some described the longing to walk outside or to look at the starry sky. Physical activities during treatment of ED are a disputed theme in existing research, as some mean physical activity is a part of the illness, and therefore do not want to implement it in treatment (Bratland & Skogmo, 2011). Nevertheless, physical activity can for some be a method for reducing/managing challenging thoughts/feelings (Trangsrud et al., 2020). A new study shows that patients with ED experienced the same treatment-effect by receiving training- and diet guidance, as they did by cognitive therapy after 6–12 months (Mathisen et al., 2020). Bratland and Skogmo (2011) argue for a focus on the joy of motion, and not on the burning of calories. At the same time, patients in treatment where physical exercise is included can get to know their bodies better, have guidance then when it comes to training and food - amount and be helped in situations where misinterpretations can be corrected (Bakland et al., 2018).

Patients mostly described a wish to experience being something other than a patient, something they often attributed to participation in different everyday activities - not necessarily workouts.

Knowing that health-promoting measures, increased mastery and wellness, can contribute to an increased engagement for the long-term recovery process (McArdle & Sheridan, 2016), our results underline the need to facilitate activities that can increase motivation during recovery. This could further contribute to developing an identity separated from the illness and be of meaning for patients like the participants during treatment (Federici & Kaplan, 2008). Based on previous research facilitating activities could also contribute to experience of autonomy, something that is shown to be of meaning for patients’ engagement in processes of change (Darcy et al., 2010). We interpret that responsibilities and having opinions increased the participants' motivation during treatment.

Both our results, as well as existing research, show the importance and necessity to focus on areas of mastery to reduce challenging feelings/thoughts and contribute to a more meaningful process of change towards identities separated from the illness.

### Letting go of the ED

The participants described it extremely difficult to let go of the ED. Some tried to motivate themselves to fight towards recovery. *“It´s important to remind myself that I am me, not an ED”.*

Mostly the grief and ambivalence that were experienced during treatment was expressed by the participants:

*Grieving that I let go, and haven´t quite seen the meaning of it today. It´s easier to turn to the ED and not deal with this, but if I´m going further with this and want answers, I have to deal with it*

*I´m mourning over that I have to let go of the ED, because I haven’t seen the meaning of it today.*

The participants described how their behaviour was influenced by the ED, and expressed how they could experience a sense of control by maintaining this behaviour.

*I acted destructively after lunch, I threw up trying to breathe and punish myself. I also threw up after dinner because the thought of allowing myself food was too overwhelming.*

*I then distract myself/escape from the thought of allowing myself food and other underlying thoughts, feelings and memories this contributes.*

Destructive behaviour was also described to have some kind of positive psychological effect as it reduced inner tension and distracted challenging thoughts or emotions.

*I want to release the pressure, find comfort and punish myself (satisfying) by throwing up, starving or harming myself.*

*I have derived reminders and concerns by not nurturing it and focusing on something else as control of something as specific as food and eating- pattern.*

They seemed to feel guilt and an experienced need to compensate for trying to distance themselves from behaviour characterized by the ED.

*Today I switched dinner with a tasteless bread meal, because it was really hard to treat myself with food- especially food I like.*

*I felt the need to compensate by reducing the amount of the last meal of the day, choosing something I don’t like to eat and season it way too much.*

*It`s mental, I can`t experience something tasting good. I then feel discouraged, and it feels meaningless.*

The treatment process seemed like an ongoing battle within themselves, because in general they wanted to become healthy.

*I`m so tired of having an ED, it`s not a life.*

*This has to be the last time, the ED can`t take any more years from me, it has taken too many already.*

### Discussion of «Letting go»

The participants described conflicted thoughts and a need for a reminder that the ED was not defining for who they were, but they still clearly described difficulties by letting it go.

For some, the ED can be viewed as a friend (Bamford et al., 2016) because it can contribute to setting limits, simplification and attenuation of emotions (Skårderud, 2000). Our interpretation is that these participants have experienced positive functions from the ED, that helps reduce difficult feelings. The experience of positive effects on their behaviour characterized by the illness seems to be something that complicates their recovery process. Also, it seems complicated if they don`t allow themselves food they like since this could be of help. Skårderud (2000) underlines that for some patients, food regimes, and thought patterns are affected by allowed/forbidden food or specific quantities.

In our interpretation it seems like the ED has a limiting function for their lives. The literature shows that behaviour characterized by the ED/compensatory behaviour can give a sense of control (Bamford et al., 2016). For example, by provoked vomiting or fasting (Knudsen et al., 2009). Our analysis questions if the participants experience positive functions of the ED, it is possible that this brings them a feeling of safety. Further, they may feel that this safety is threatened because these functions are tried to be gradually separated from them during treatment.

Due to the positive functions the participants could be experiencing from the ED, we interpret that it may feel safer for them to hold on to it than letting it go, especially if letting it go contributes to challenging feelings.

## Summary

The study´s results shows that the days in treatment are mostly characterized by difficult experiences where challenging and contradicting feelings and thoughts were commonalities. Not even one of the 14 days the participants wrote diaries contained expressions without such experiences. These challenges regarding their experienced difficulties of meals in general, a “week of considering”, lack of everyday activities, different maintenance of rules by the health staff, longing for life outside of treatment and letting go of the ED. On the other hand, experiences that contributed to meaning during treatment were conversations (the health staff availability, guidance and advice), individual- and group activities, having responsibility and meals with others.

Because the participants clearly described more physical and psychological challenges than experiences that were of positive meaning during treatment, we identified a call for more meaningful/everyday-like experiences during the days spent at in hospitalized treatment, to increase the patient’s motivation. This is done by facilitating activities that can contribute to experienced mastery besides the ED, where their physical and physiological needs are still met. Further, as our results clearly show, this could contribute to increasing their feelings of self-worth and learning how to cope with triggering challenges in other ways than taking control over food and/or self-harming.

### Methodological considerations

The method of data collection seemed to be of help for the participants, as they described that the diaries made it possible to put thoughts and feelings into words. Further, this method could contribute to self-determination, because the participants chose what they wanted to write, without any influence from the researchers. The study’s data material is limited but valuable because it shows participants own descriptions of experiences during two whole weeks of treatment.

Using an interpretative analysis method of the written narratives was experienced valuable by the researchers, regarding the wish to highlighting patient’s voices. This method made it possible to get a new perspective on treatment experiences, by having a close view to immediate descriptions during the treatment context and using firsthand experiences in the presentation of the study’s results. If the researcher had chosen a different method for collecting data, as for example, interview, it would have been possible to ask the participants if the researcher had understood their descriptions correctly. Therefore, when using diaries for data collection, the participants’ quotes were used contributing to the study’s credibility.

When it comes to reliability, this study cannot be replicated the exact same way, because of the researcher’s perspectives that can be changed over time (Holloway, 2008). Regardless, if another researcher has used the same material and approach, they could have acquired more or less the same understanding and some equivalent results. Especially if they have used Skårderud’s frame of understanding, as this study has, considering that he is an internationally recognized specialist of ED. The participant’s descriptions can also be changed, for example, in relation to which part of the treatment process they are in mood or different timing.

In this study, the researchers described the approach and methods that contribute to validity (Holloway, 2008). Also, the study´s results reflect the purpose of the study. Before starting this study, the researcher didn't succeed on finding any studies regarding patients’ experience over time in treatment for severe ED. The study has some limitations when it comes to the results. Because of the limited timeframe, the study had 3 participants. If the researcher had more time, it may have been possible to recruit more participants. This study will not show how most people with ED experience in-hospital treatment. Nevertheless, this study contributes to validity, where the results are valid for this study’s selection.

## Conclusion

**Figure 1.**
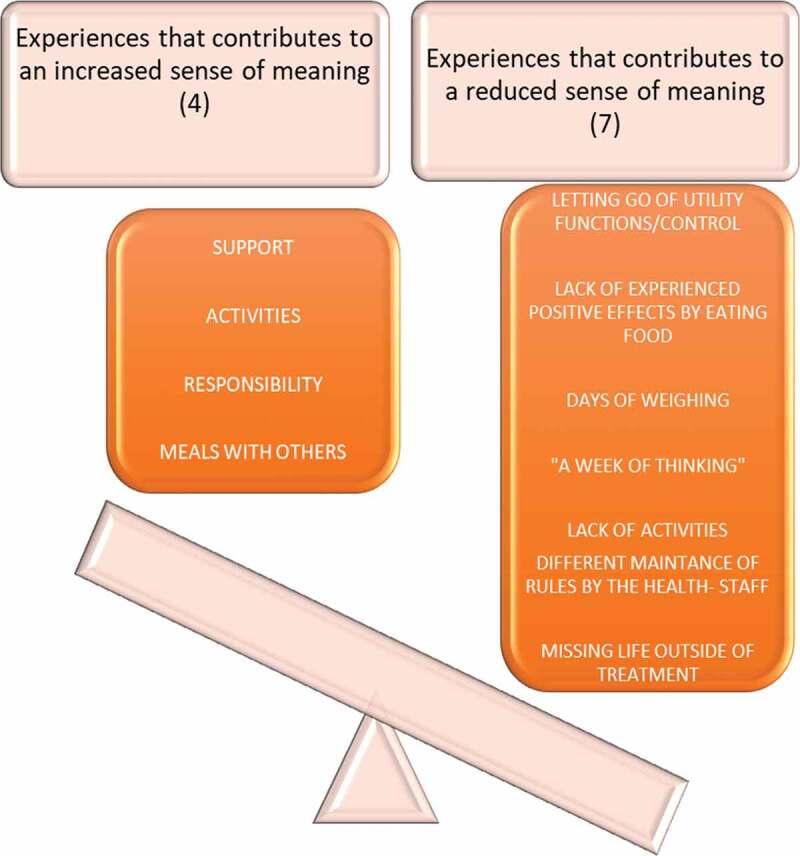
Visual presentation of the study’s results, which shows the balance of experiences that contributed to increased/reduced sense of meaning, and is likely to influence the participants motivation (Storli, 2018)

The study has shown that days of treatment are mostly characterized by experiences that might impact the participants' sense of meaning, and likely their motivation during the recovery process (Figure 1). Because this study points at the importance of new arenas of mastery, this is something that ought to be further researched in the future. This is in relation to how such arenas can increase meaningful experiences and motivation during treatment and reduce focus on their body and food. Further, it would be interesting to study if this could contribute to an increased engagement, and maintenance of changed behaviour- patterns after treatment. For future treatment, this study suggests that mental healthcare on facilitating treatment that may contribute to increased meaning and motivation by identifying expressed needs and supporting new ways of managing their ED in everyday life. Thus, conveying belief in the individual persons and offering activities that that provide a safe environment to develop a new identity as young persons without illness.
